# The Link between the Perception of Animal Welfare and the Emotional Response to Pictures of Farm Animals Kept in Intensive and Extensive Husbandry Systems: An Italian Survey

**DOI:** 10.3390/vetsci10110652

**Published:** 2023-11-13

**Authors:** Giacomo Riggio, Elisabetta Angori, Laura Menchetti, Silvana Diverio

**Affiliations:** 1 Laboratory of Ethology and Animal Welfare (LEBA), Department of Veterinary Medicine, University of Perugia, Via San Costanzo 4, 06126 Perugia, Italy; giacomoriggio@gmail.com; 2 Independent Researcher, Via IV novembre 13, 52044 Camucia, Italy; ely--@live.it; 3 School of Biosciences and Veterinary Medicine, Camerino University, Via Circonvallazione 93/95, 62024 Matelica, Italy

**Keywords:** emotions, livestock, outdoor, indoor, poultry, chickens, pigs, cows, rabbits, consumer

## Abstract

**Simple Summary:**

The number of animals bred to keep up with the increasing demand for animal food products is steadily growing. Different husbandry systems often guarantee different animal welfare standards, which may condition people’s choices as animal product consumers. In this study, we explored people’s emotional responses toward pictures of farm animals (cows, pigs, chickens, and rabbits) kept in intensive and extensive husbandry systems and how it related to their perception of animal welfare as well as their food choices. A total of 835 respondents completed the questionnaire. As expected, pictures of animals in intensive systems elicited negative emotions, especially for pigs and rabbits, whereas pictures of extensive systems, elicited positive emotions, especially for chickens. Intensive systems were perceived to guarantee lower animal welfare levels. Regardless of the husbandry system, cows were perceived to have the highest welfare levels. Most importantly, the quality of the participants’ emotional responses was positively associated with the perception of animal welfare and negatively associated with the importance given to welfare when purchasing animal products. Furthermore, several demographic factors, namely gender, education, household composition, living area, pet ownership, and eating habits were found to affect the participants’ emotional response to farm animal pictures.

**Abstract:**

As livestock production grows to satisfy the global demand for animal products, understanding public attitudes towards different husbandry systems becomes essential for both animal welfare and socio-economic reasons. This study aimed to investigate people’s emotional responses toward pictures of farm animals kept in intensive and extensive husbandry systems, their perception of animal welfare, and their choices as animal product consumers. A questionnaire that included demographic questions and photos of cows, pigs, chickens, and rabbits in both intensive and extensive systems was distributed electronically and physically and completed by 835 respondents. Photos of animals in intensive systems elicited more negative emotions, especially for pigs and rabbits (*p* < 0.05), as opposed to extensive systems, which elicited more positive emotions, especially for chickens (*p* < 0.001). Higher welfare levels were perceived for extensively farmed animals (*p* < 0.001) and for cattle compared to all other species, regardless of the husbandry system (*p* < 0.001). The quality of the emotional response was positively associated with welfare perception (*p* < 0.001) and negatively associated with the importance given to welfare when purchasing animal products (*p* < 0.001). Finally, the emotional response was found to be affected by gender, education, household composition, living area, pet ownership, and eating habits. The implications and limitations of these findings are discussed.

## 1. Introduction

In 2021, approximately 73 billion chickens, 330 million cows, 1.5 billion pigs, and 1.3 billion rabbits were slaughtered for food production worldwide [1]. As high as they may seem, these numbers are destined to rise in order to keep up with an ever-growing global demand for animal proteins [2,3]. Indeed, such a high production is mainly achieved via intensive farming. Unfortunately, the type of management that often characterizes intensive husbandry systems has been reported to jeopardize the psychophysical welfare of the animals, regardless of the species [4,5,6,7,8,9,10,11,12,13,14,15,16]. Although different husbandry systems may have their own specific risks in terms of animal well-being (e.g., increased incidence of parasitic diseases in organically farmed animals [17]), it is widely accepted that extensive and organic farming provides animals with a social and physical environment more suitable for guaranteeing higher welfare standards [18].

Nonetheless, as of 2019, only 4% of bovines, 1.1% of swine, and 4.2% of poultry were farmed organically in the European Union [1]. The pressure exerted by the general public on stakeholders, governments, and livestock producers, both in the form of activism and consumer behavior [19], has been a major drive to switch from intensive to extensive husbandry practices. 

Public preference for extensive and organic farming systems may be due to several reasons, including a perceived reduction in the use of GMOs, hormones, and antibiotics [20] or the higher sustainability of animal production [21]. Notwithstanding, people’s interest in farm animal wellbeing has increased over the last few decades to the point that their attitude towards different husbandry systems may now be primarily affected by their perception of the animals’ level of welfare [22,23,24,25]. 

In reality, laypersons rarely have the opportunity to assess first-hand the welfare of farm animals, and their perception and judgment of different husbandry systems is mainly, if not solely, based on images and videos reported by mass media [19,26]. In this regard, Busch et al. [27] reported that pictures of conventional broiler barns were perceived very negatively by German respondents in terms of animal health, environmental hygiene, the possibility to express normal behaviors, and overall care provided to the animals. Negative responses were also obtained in a similar study by Gauly et al. [28], where they showed people images of pig fattening and farrowing pens. In another picture-based study, Kühl et al. [29] found that the general public is much more willing to accept husbandry systems that provide the animals -specifically cows, chickens, and pigs- with outdoor access than traditional indoor-only systems. 

Interestingly, photographs of animals seem to trigger the activation of the human amygdala, which processes emotions, even more intensely than photographs of people [30]. The role of the affective dimension in processing livestock pictures is well known by marketers, farmers, media, and animal rights activists who attempt to influence people’s judgment and actions using images that elicit specific emotions [31]. In fact, according to the social intuitionist theory, our decision making is built on a first involuntary emotional reaction, which, only at a later time, we try to justify through rationalization [32].

Nonetheless, to the best of our knowledge, only one recent study directly examined people’s emotional responses to photographs of farm animals in different circumstances, namely outdoor, indoor, and suffering [31]. They found that pictures of animals kept indoors and suffering generated negative emotions, whereas those of animals kept outdoors generated positive emotions. They also found that the observer’s professional background, belief in animal sentience, and personality traits affected their emotional response to animals kept indoors and suffering. 

However, this study did not directly examine the link between emotional response, perception of animal welfare, and choices in animal product consumption. This may have ethical and socio-economic implications since previous research suggests that consumer’s behavior may be affected, among others, by their perception of animals’ level of welfare [33,34,35,36].

Therefore, the primary purpose of this study was to investigate how pictures depicting animals in different husbandry systems (intensive vs. extensive) impacted the emotional response of non-professional observers and how the latter linked to their perception of the animal’s welfare, as well as to their choices as consumers of animal products. Furthermore, we examined the effect of demographic factors of both observers and animals on the former’s emotional response to the images presented.

## 2. Materials and Methods

### 2.1. Participants

An ad hoc illustrated questionnaire was voluntarily and anonymously completed by 835 Italian residents. In order to provide a sample representing the various age groups within the population, the questionnaire was distributed both electronically, through social networks and e-mail, and physically to people in social community and commercial centers, university departments, senior recreation, and rest homes. The only exclusion criterion was being under 18 years of age. We could not calculate the relative response rate of the questionnaire because of the way it was distributed, i.e., virtual snowball sampling technique.

### 2.2. Questionnaire: “Human Emotions and Animal Husbandry”

The questionnaire, named “Human Emotions and Animal Husbandry” (Figure S1), was specifically designed to gather information on the emotions people experience when looking at photos of different animal farming systems, on their opinion on the welfare state of the animals, and on their choices as consumers of animal products. In both the electronic and paper forms of the questionnaire, a brief introduction explained the purpose of this study. The questionnaire was divided into three sections:

Section A included 9 multiple-choice questions on demographic information (gender, age, education, residence, employment, number of family components, presence of animals at home, owning animals, vegetarian/vegan choice). 

Section B consisted of 11 photos depicting four species of domestic farm animals kept under extensive (n = 5 photos) or intensive farming (n = 6 photos), i.e., four cattle photos (two intensively reared: calves tied on a chain, mechanical milking of dairy cows; two extensively reared: a dairy cow with calf on pasture, dairy cows grazing), three of poultry (i.e., high-density indoor broilers, high density indoor laying hens, extensive chicken farm), two of pigs (one intensive and one extensive pig farm) and two of rabbits (one intensive and one extensive rabbit farm). The photos were randomly chosen out of the 20 first copyright-free images appearing on Google after using species (i.e., rabbit, cow, pig, chicken) + husbandry system (i.e., intensive, extensive) as search keywords. Participants were asked to observe each photo and express their emotions and opinions on the animals as follows:

- Assigning a score to rate the intensity of ten emotions (anger, joy, sadness, surprise, shame/disappointment, resignation, hope, nostalgia, remorse/guilt, contempt/disgust) felt while watching the photo using a 5-point Likert-type scale (where 0 was the equivalent of an emotion unproven and 5 was the maximum intensity of the emotion felt);
- Reporting their opinion on the level of welfare they attributed to the animals shown in the photo using a 5-point Likert-type scale (very poor, poor good, excellent, not known).

Section C consisted of 4 multiple-choice questions investigating how much the respondents considered “animal welfare” important when they purchased animal products, their weekly consumption of poultry, beef, pork, and rabbit meat, milk, and eggs, as well as if they were vegetarian or vegan, and in this case, the reasons for their choice.

For each photo, rating scales were used to allow the respondents to express their opinion on the level of animal welfare (Animal Welfare score: from 0 = “bad” to 5 = “excellent”) and the importance of animal welfare when purchasing animal products (Welfare Importance score: from 0 = “not important at all” to 5 = “very important”) [37].

At the end of the questionnaire, the respondents could freely write comments and suggestions on issues concerning animal welfare and farming. Overall, the questionnaire was straightforward and required only a few minutes to be completed.

### 2.3. Statistical Analysis

The data obtained from the questionnaires using Google Forms^®^ were entered into an Excel spreadsheet and transferred into the statistical program SPSS Statistics version 23 (IBM, SPSS Inc., Chicago, IL, USA) for analysis. The level of statistical significance was set at <0.05. The distribution within the categorical variables was analyzed using Chi-Square Goodness-of-Fit tests. 

The participants’ emotions expressed for photos in Section B were analyzed using the Principal Component Analysis (PCA) with Varimax rotation. The variables to be included in the PCA were selected in accordance with the correlation matrix. The number of components was chosen according to eigenvalues and according to screeplot. The internal consistency was assessed using Cronbach’s alpha. Thus, the PC Emotion variable was created as a linear combination of selected variables. The component’s score was determined using the regression method. Then, the PC Emotion variable was analyzed using Mixed linear models, including participants as subjects and photos as repeated factors. Sidak correction was used for multiple comparisons. First, we evaluated the effect of the photo features, i.e., type of husbandry (intensive vs. extensive) and species (bovine vs. swine vs. poultry vs. rabbits) depicted in the photo. Then, we built multivariable models to evaluate the influence of participants’ demographic data on the PC Emotion stratified by type of husbandry and including species as covariate.

Questions of Section C were treated as an ordinal variable and analyzed using Generalized Estimating Equations procedure with cumlogit link function and multinomial distribution. For Welfare scores analysis, participants and photos were included as subjects and repeated factors, respectively. Results were expressed as odd ratio (OR) with corresponding 95% confidence intervals (CIs) and *p* values (from Wald statistics).

## 3. Results

### 3.1. Participants Demographics and Their Eating Habits

The majority of the participants were women (57.4%, χ^2^ = 18.1, *p* < 0.001), aged less than 25 years (28.7%, χ^2^ = 12.1, *p* < 0.01), with a high school diploma (43.1%, χ^2^ = 299.8, *p* < 0.001), resident in villages with up to 5000 inhabitants (45.3%; χ^2^ = 109.8, *p* < 0.001). Most of the questionnaires were compiled by students (39.4%, χ^2^ = 921.7, *p* < 0.001), and most of the participants had a family composed of more than two members (58.0%; χ^2^ = 234.2, *p* < 0.001). Only 23.5% of the respondents did not own any animal (χ^2^ = 235.0, *p* < 0.001), but among these, 80.8% (χ^2^ = 47.4, *p* < 0.001) declared they had lived with an animal in the past (Table S1). The majority of the participants consumed meat of bovine (χ^2^ = 1415.2, *p* < 0.001), meat of pork (χ^2^ = 1120.0, *p* < 0.001), and eggs (χ^2^ = 1413.6, *p* < 0.001) up to two times a week, while never ate rabbit meat (χ^2^ = 1657.2, *p* < 0.001). Most of the participants also consumed poultry meat up to two or four times a week (χ^2^ = 484.8, *p* < 0.001). Milk was consumed either never or every day by most of the respondents (χ^2^ = 343.4, *p* < 0.001), whereas only a few of them reported intermediate consumption frequencies (Table 1). 

In addition, only 8.3% (69 people) of the 835 participants reported being on a vegan or vegetarian diet (χ^2^ = 581.8, *p* < 0.001). This vegetarian/vegan decision was largely motivated (98.6%, *n* = 68, χ^2^ = 65.1, *p* < 0.001) by an ethical choice. Only one vegetarian/vegan reported they made this choice because they did not like meat (1.4%) (Table S1).

### 3.2. Human Emotions: The PC “Emotion Quality Axis”

Data collected in Section B were processed using the Principal Component Analysis (PCA) to reduce the number of variables and to synthesize the emotions with one or a few scores for each photo. Initially, all the emotions (*n*° = 10 items: anger, joy, sadness, surprise, shame/disappointment, resignation, hope, nostalgia, remorse/guilt, contempt/disgust) were introduced into the PCA. Following the analysis of the correlation matrix, the emotions “nostalgia” and “surprise” were excluded because they were little correlated to the others. Therefore, eight items remained in the PCA that produced a single component, an Emotion Quality Axis, hereby called PC Emotion, for clarity. The PC Emotion explained 69.5% of the total variance and had an acceptable internal consistency (Cronbach’s alpha = 0.687).

**Figure 1:**
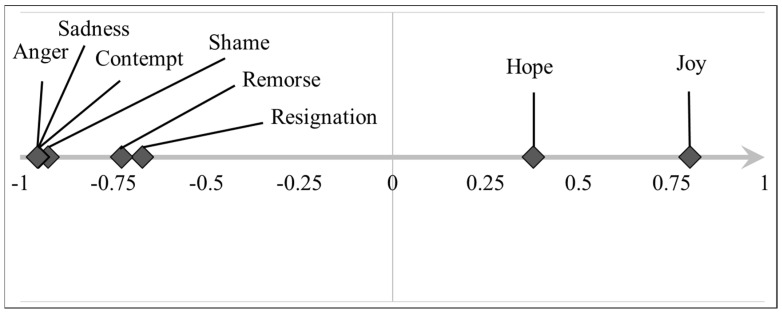
Graphic representation of the PC emotion. The diamonds indicate the loadings of each item as extracted using the Principal Component Analysis. The PC Emotion explained almost 70% of the total variance and had an acceptable internal consistency (Cronbach’s alpha = 0.687).

The PC Emotion extracted expresses the degree of anger–joy, with positive loadings for negative emotions (anger contempt/disgust, sadness, shame/disappointment, remorse/guilt, resignation) and negative loadings for positive emotions (joy and hope). To facilitate the interpretation of the PCA component, the sign of the loadings was reversed so that a negative loading of the PC Emotion indicated intense emotions of anger (loading = −0.954), sadness (loading = −0.950), contempt/disgust (loading = −0.950), shame/disappointment (loading= −0.925), remorse/guilt (loading = −0.727), and resignation (loading = −0.672), while a positive loading indicated joy (loading = 0.800) and hope (loading = 0.379) (Figure 1).

Therefore, the new variable, created via the scoring of the PC Emotion, associated positive values for photos where the emotions of joy and hope prevailed and negative values where emotions of anger, sadness, remorse/guilt, resignation, contempt/disgust, and shame/disappointment were expressed.

### 3.3. Influence of Farming Systems and Animal Species on Human Emotions

The first model highlighted the strong influence on the PC Emotion of the farming system (F = 21704.5, *p* < 0.001). As shown in Figure S2, photos depicting animals kept under intensive systems had a lower score (more emotions of anger, sadness, remorse/guilt, resignation, contempt/disgust, and shame/delusion) than those kept in extensive farms (more emotions of joy and hope). Subsequent analyses were performed stratifying by type of farming (separately in intensive and extensive farms).

**Figure 2:**
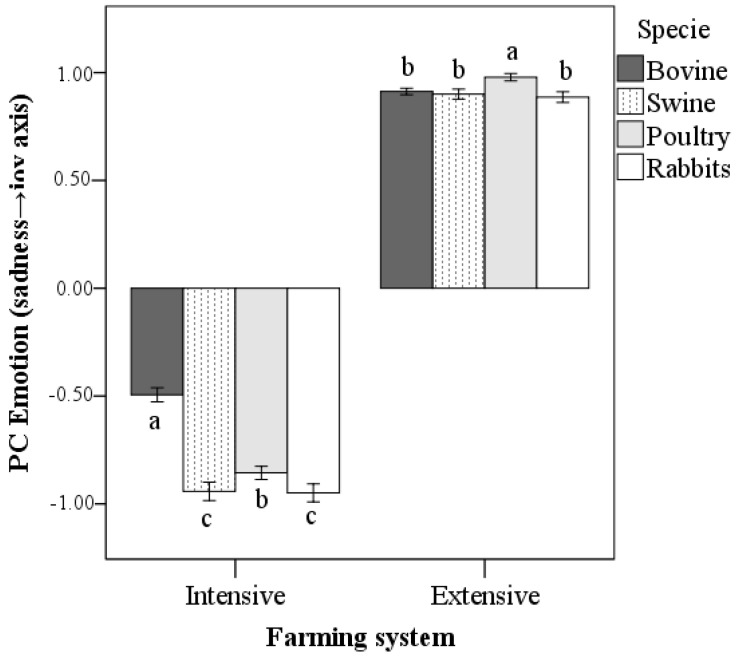
PC Emotion scores obtained in intense and extensive farming in accordance with the species depicted in the survey’s picture (mean ± 95% CI; bars of each type of farming system that share the same letter are not significantly different at the level of 0.05; Sidak correction).

Specie differences in PC Emotion score were found for both intensive (F = 151.2, *p* < 0.001) and extensive husbandry (F = 13.5, *p* < 0.001). For intensive farms, the score of the PC Emotion was higher (i.e., less negative) for photos depicting bovines than other species (*p* < 0.001). The lowest (i.e., the most negative) scores were obtained for pigs and rabbits (*p* < 0.05; Figure 2). For extensive farming, higher scores were obtained for photos of poultry (*p* < 0.001), while there were no differences between the other species (Figure 2).

### 3.4. Influence of Demographic Factors and Eating Habits of the Participants on Human Emotions

Compared to female participants, males had higher scores both for photographs depicting intensive (F = 4.0, *p* < 0.05) and extensive farms (F = 11.0, *p* < 0.01). Participants with a university degree scored higher photos representing extensive farming compared with those with middle and higher school diplomas (F = 5.4, *p* < 0.01). Participants living in cities had a less negative perception of intensive farming, giving higher scores compared with participants living in villages (F = 3.2, *p* < 0.05); they also had the lowest scores in extensive farming (F = 6.0, *p* < 0.01). Regarding the results concerning the number of family members, participants with a family unit composed of a single person had the most negative PC emotion scores for photos of intensive farms (F = 8.4, *p* < 0.001). The presence of animals in the house determined more negative PC emotion scores for photos of intensively farmed animals (F = 23.7, *p* < 0.001), while participants who had animals in the past had the highest score for extensive farming (F = 40.1, *p* < 0.001). The age and job of the participants did not affect any score. 

Finally, the PC Emotion scores were analyzed according to the eating habits of respondents: vegetarians achieved lower scores than non-vegetarians in both intensive (F = 5009.0, *p* < 0.001) and extensive husbandry photos (F = 265.1, *p* < 0.001). A summary of these results can be found in Table 2.

### 3.5. Animal Welfare Perception and Human Emotions

For each photo, participants were invited to express their opinion on the level of animal welfare (Welfare score). The highest Welfare score indicated better levels of perceived welfare. Extensive farming obtained higher Welfare scores than intensive farming (OR = 250.6, 95% CI = 212.0–296.3, *p* < 0.001). Holding the type of husbandry constant, cattle had higher welfare scores compared to swine (OR = 0.3, 95% CI = 0.3–0.3, *p* < 0.001), poultry (OR = 0.4, 95% CI = 0.4–0.5, *p* < 0.001), and rabbits (OR = 0.3, 95% CI = 0.3–0.4, *p* < 0.001). Welfare scores were positively associated with PC Emotion (OR = 22.0, 95% CI = 19.1–25.4, *p* <0.001; Figure S3), so the higher the level of welfare perceived in the photos, the greater the positive feelings were reported. Finally, vegetarian participants attributed a lower Welfare score than non-vegetarians (OR = 0.5, 95% CI = 0.5–0.6, *p* < 0.001).

### 3.6. Importance of Animal Welfare on Food Choices and Human Emotions

For most of the respondents (χ^2^ = 791.1, *p* < 0.001), animal welfare was very important (*n* = 450, 55.1%) or important (*n* = 222, 27.2%) when purchasing animal products. Animal welfare was moderately important (*n* = 94, 11.5%), of little importance (*n* = 46, 5.6%), and not important at all (*n* = 5, 0.6%) for the rest of the participants.

**Figure 3:**
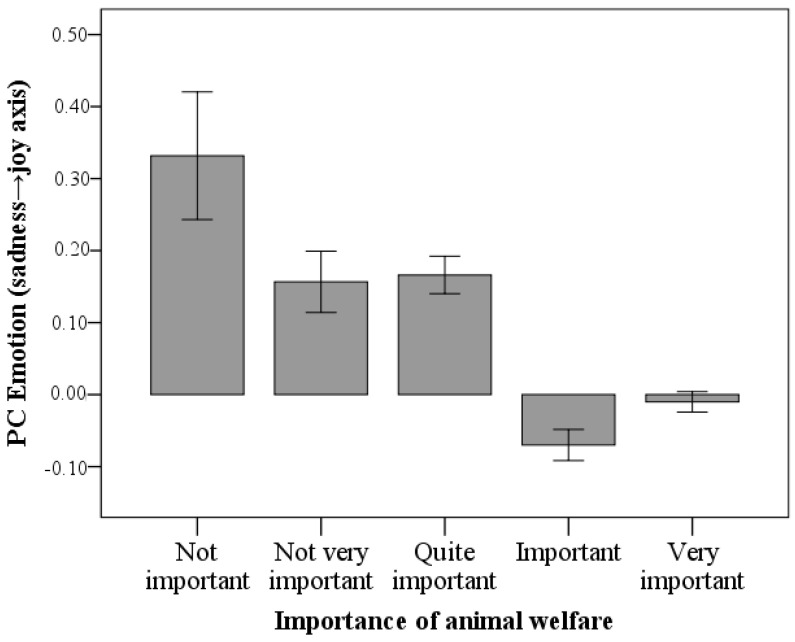
Association between importance that participants attributed to welfare conditions during the purchase of animal products and mean score of PC Emotion.

A negative association was found between the importance of animal welfare when purchasing animal products and the mean PC Emotion score of each participant (OR = 0.4, 95% CI = 0.3–0.7, *p* < 0.001) so that the respondents that attributed greater importance to animal welfare in their food choices also had an emotional response to the photos characterized by higher levels of anger and sadness (Figure 3).

## 4. Discussion

### 4.1. Husbandry Systems, Animal Welfare and Human Emotions

As expected, the type of husbandry system had an impact on the emotional response of the participants. Regardless of the species, the sight of animals in intensive husbandry systems generated negative emotions, whereas animals being bred extensively aroused emotions of joy and hope. This result is in agreement with several previous studies reporting public perception to be in favor of extensive rather than intensive farming [38]. There may be various reasons behind people’s general preference for extensive farming systems [39]. Animal welfare can be one of them, as suggested by both higher perceived welfare scores for animals kept in extensive systems and the correlation between welfare scores and quality of emotional response in this present study. While there is scientific evidence associating intensive husbandry practices with lower levels of psychophysical animal welfare [40,41], it is very rare for the general public to have their opinion built on direct observations of animals’ psychophysical conditions within the farming system. Most of the time, people’s opinion on this subject is formed via images and videos broadcasted by mass media, which tend to create a narrative of positive extensive versus negative intensive farming [38]. However, a few studies showed that people’s perception of livestock breeding can change, for the better or for the worse, after personally witnessing how farm animals are managed (e.g., on-farm tours, open days for the public) [42,43]. Unfortunately, we did not ask our participants whether they had this type of experience, but it would be interesting for future studies to investigate whether it may affect their emotional response to livestock images. 

In our study, the species of the animal affected the participants’ emotional responses. More specifically, amongst the pictures portraying intensive farming systems, the one with bovines exerted the least negative emotional response. In fact, scores for bovines were lower than for all other species in each negative emotion addressed in the survey. Similarly, Kühl et al. [29] found that indoor housing for cows is more positively accepted by German citizens than for pigs and chickens. One possible explanation is that in Europe, people have many more chances to see large-scale extensive farming systems with cows (e.g., when they are grazing) rather than with commercial pigs, chickens, and rabbits. Such experiences may positively affect the feelings people have towards the cow breeding sector while remaining suspicious in the case of less visible species [44]. This may be especially true in the case of our study, where the respondents were not given any explanations on the content of the photo, which may have also portrayed a restrictive practice put only temporarily in place (i.e., calf tied to a short chain, dairy cows physically restrained during milking). Regardless of the specific motivation, the lower negative emotional response is most likely due to the higher perceived level of welfare reported for this species in our study. Surprisingly, the fact that rabbits may also be kept as pets did not seem to affect either people’s perception of their level of welfare or the quality of their emotional response to the pictures, which were similar to those reported for other species. One possible explanation is that the culture of keeping rabbits as pets in Italy is far more recent than and not as widespread as in other European countries [45,46]. This may be reflected in people’s perception of this species mainly as a farm rather than a companion animal. 

As for extensive farming, pictures of chickens induced the most positive emotional response. This may be due to the more intense feelings of surprise and hope reported for this species in our study. Possibly, the surprise is linked to the public’s lack of awareness that commercial chicken breeding can be of an extensive type, as well. Instead, hope may signify that the photos induced the respondents to wish for a change in poultry farming. Although this interpretation is supported by previous findings that the general public shows the highest concern for and need for a change in the treatment of laying hens and broilers [44,47,48] amongst all farm animals, it remains highly speculative and should be more deeply investigated.

### 4.2. Socio-Demographic Factors, Animal Welfare, and Human Emotions

Overall, female respondents were more negatively affected by pictures of intensive farming and less positively affected by pictures of extensive farming compared to males. This result is absolutely in line with the scientific literature, which consistently reports females show a greater tendency to consider non-human animals as sentience beings [49], to express higher concern for animal welfare issues [50,51], and to be less tolerant towards the use of non-human animals for human benefits, including breeding for food [52]. Emotions play a central role in shaping this gender-based distinction in people’s attitudes towards animals. As a matter of fact, women appear to be more empathetic towards non-human animals; that is, they are more capable of understanding and sharing the animals’ feelings and emotional states. Whether this enhanced feminine capability is due to socio-cultural [53] or biological [54] factors is still a matter of debate [52]. Regardless, it is likely that the female respondents in our study were more strongly affected than their male counterparts by the animals’ perceived emotional state.

As for education, participants with a university degree reported a more positive emotional response to pictures of extensively farmed animals compared to participants with lower education levels. In support of this result, a series of studies by Kellert [55,56,57] consistently found that higher education was associated with greater interest and appreciation for animals and nature.

Respondents from urban areas were less negatively affected by intensive farming pictures, as well as less positively affected by extensive farming pictures, compared to respondents living in small villages. This finding is in contrast with the general notion that people from the city tend to show greater concern for animal welfare issues [23,58]. However, it is likely that people’s emotional response is not solely based on their perception of animal welfare. In fact, the scoring pattern of urban citizens seems to point towards an overall reduced emotional involvement rather than simply a reduced sensitivity towards animals (i.e., less negative impact of intensive farming and more positive impact of extensive farming). Possibly, in urban areas, people have undergone a process of detachment from animals and the natural world [58,59,60], which may affect their level of emotional connection with those species that are not an integral part of their city life. On the contrary, people in the countryside may still have the chance to experience a stronger physical and emotional connection with farm animals and also express greater appreciation for certain (i.e., extensive) husbandry methods as part of their rural culture [61], regardless of their impact on animal welfare. 

Respondents who were single were more negatively affected by the pictures of animals in intensive husbandry systems compared to those living in households composed of more than one individual. This result is in line with previous studies that report married couples and couples with children to be less supportive of animal rights [53] and less emotionally attached to animals [62]. 

Current and past pet ownership were associated with a more negative emotional response to intensive farming and a more positive emotional response to extensive farming pictures, respectively. While it is hard to speculate on these specific differences, it seems clear that, in our study, taking care of a pet resulted in deeper empathy towards farm animals, as well. This is in line with the “Pets as ambassadors hypothesis” proposed by Paul and Serpell [63], which theorizes that the affective nature of our interactions with pets may reverberate in the quality of our perception and attitude towards a broader range of animal species [64]. 

With regard to livestock, Boogaard et al. [65] found that pet ownership strongly contributes to a less positive perception of farm animals’ quality of life. Generally speaking, the positive effect of pet ownership on people’s attitudes toward animals appears to be a constant and straightforward finding across anthrozoological studies [66,67,68,69,70].

### 4.3. Food Choices, Animal Welfare and Human Emotions

The proportion of respondents following a vegetarian or vegan diet in our experimental sample (8.3%) is consistent with that reported in Italy at the time of the study (7.6%) [71]. The emotional response pattern of vegetarians versus non-vegetarian respondents is similar to that found for females versus males, with a higher negative emotional impact for intensive farming and a lower positive emotional impact for extensive farming. Therefore, we can make a similar inference that vegetarians are more empathetic than non-vegetarians towards non-human animals [72]. Again, the perception of the animals’ level of welfare seems to play a mediating role in the quality of the emotional response since vegetarians scored significantly lower for animal welfare than non-vegetarians. It should be noted that although a vegetarian diet may be undertaken for different motivations that do not necessarily imply concern over animal welfare issues, in the case of our study, the majority of the respondents reported that their choice was due to ethical reasons. Overall, these were expected findings since there is a quite vast body of literature describing the reduced tolerance of vegetarians towards the use of animals for human benefits [73], their higher concern for animal welfare issues [74,75], and their deeper empathy towards non-human animals [76]. 

Finally, regardless of whether a vegetarian diet was followed, people who reported a higher negative emotional response to the photos of the animals also indicated animal welfare to be more important when choosing what food products to purchase. This result is in line with previous findings reporting that consumers may modify their food purchase behavior according to their interest in animal welfare issues [77,78]. However, other studies found that greater concern for animal welfare issues does not always translate into higher demand for food products that guarantee higher animal welfare standards [79], especially when higher prices must be paid [80]. Hence, it is possible that the self-reported greater importance given to animal welfare when purchasing food may not necessarily reflect a final decision in favor of more “animal-friendly” products. These doubts may only be resolved by using more objective sources of information on people’s food purchases rather than personal statements [39]. Generally speaking, we suggest that investigating the emotional impact of the perceived levels of animal welfare rather than just the perception of animal welfare may help explain why, in some people, their interest in animal wellbeing does not reflect a change in food purchase behavior. 

This study has multiple limitations. First of all, the questionnaire was distributed online; therefore, participation was on a voluntary basis. This may create a problem of self-selection bias because people who decide to participate in this study are likely to have a higher interest in the topic of this research—in this case, farm animal welfare—than those who decide not to be involved. This may also explain why almost half of the respondents resided in rural areas, although the algorithm used by social media platforms may also have played a role in how the questionnaire circulated online. Common evidence of self-section bias in animal welfare research is the greater involvement of women compared to men [81,82]. However, in this current study, the proportion of female/male respondent ratio was almost even, suggesting that this phenomenon may have occurred only marginally. On the contrary, our sample appeared biased towards students and young respondents (less than 25 years old). Again, this has likely been caused by the distribution means used to collect responses. On the one hand, some social media may be more actively used by young people, although this may not be the case for Facebook^®^ [83], which was the social platform used to publicize this survey. On the other, because of the algorithms used, they tend to share the content posted online (e.g., the link to our questionnaire) with users with similar characteristics and interests, affecting the heterogeneity of the experimental sample [84]. 

Future studies may opt for different methods of distribution (e.g., on-paper distribution, social media with less polarizing algorithms), which may decrease the absolute number of responses but may improve the generalizability of the results.

Questionnaire-based studies investigating people’s opinions on sensitive topics with ethical implications, such as farm animal welfare, may trigger a social desirability bias. Social desirability is the tendency of individuals to present themselves in socially acceptable terms [85]. This may create a distortion of their responses toward socially acceptable traits, values, attitudes, and opinions [85,86]. This type of bias plagues research based on self-reports, and it should always be taken into account when interpreting results [86]. Although we do not have evidence of that, the unbalanced proportion of negative versus positive attributes listed in our questionnaire for the participants to describe their emotional response may have intensified the effect of this bias even further. We can only suggest that future studies rely on techniques specifically aimed at eliciting truthful answers to sensitive questions, such as the List Experiment, which has been recently applied to survey studies in the field of farm animal welfare [87]. 

Another important limitation is that the perspective of the photo was not always the same across species. This may have conditioned people’s awareness of certain salient elements within the pictures. For instance, Busch et al. [88] found that space allowance was rated more positively when photos of a pig pen were taken from a bird’s eye perspective rather than from a human or animal’s perspective. The photos we presented were not as different in terms of perspective, rather certain aspects of the background setting, such as type of flooring, animal density, and overall space, were more visible in some pictures than others. Since these elements have been shown to affect people’s perception of animal welfare [89], it is possible that the lack of standardization of the pictures’ background also had an impact on our participants’ emotional responses. 

Not less importantly, we did not account for the technical properties of the photos that have been previously reported to alter people’s emotional response to them, such as color tone, hue, brightness, and saturation [90,91].

While it may be practically impossible to control each and every photo-related factor that may affect people’s emotional response to the picture of an animal, future studies should try to minimize their impact, possibly with the help of AI photo editing. 

Asking questions about consumer behavior and eating habits after having presented the farm animal pictures may have led the respondents to under-report their consumption of animal products and/or overstate their attention to animal welfare [92]. This may occur especially in individuals more susceptible to animal welfare issues [93]. However, the opposite is also possible, where meat eaters may have under-reported the salience of their emotional response to the pictures and/or their concern for animal welfare [94] to morally justify their dietary practices and reduce their cognitive dissonance [95]. Hence, the effect of these cognitive strategies on moral justification should be taken into account when interpreting our findings.

Finally, our study was conducted on Italian citizens only. Since people from different countries may have different ethical approaches to animals and different perceptions of animal welfare, our findings may not necessarily apply to people with different cultural backgrounds.

## 5. Conclusions

In our society, people’s perception of farm animals’ welfare, as well as attitudes towards different husbandry systems and practices, are mainly shaped by multimedia material (i.e., photos, videos) rather than direct observation and experience. Pictures of farm animals in different husbandry contexts are often used by mass media to elicit specific emotional responses in the general public. Our findings suggest that the quality of people’s emotional response to pictures of different farm animal species is strictly linked to the perceived level of animal welfare. Furthermore, both perceived animal welfare and the quality of the emotional response differed between intensive and extensive husbandry systems, with the former obtaining lower animal welfare scores and eliciting more negative emotional responses. Demographic factors, such as gender, education level, living area, household composition, pet ownership, and eating habits, appeared to have an effect on the participants’ emotional responses. Finally, the quality of the emotional response when looking at the pictures of farm animals was associated with the importance given to animal welfare when purchasing food products. Despite its limitations, this study provides new important information to understand the link between people’s perception of animal welfare, their emotional response to farm animal husbandry, and their attitude as consumers of food products.

## Figures and Tables

**Table 1:** Weekly amount of consumption of the main food of animal origin of participants.

Food of Animal Origin	Weekly Consumption			
	Never	Up to Two Times a Week	Up to Four Times a Week	Every Day
Bovine meat	124 (15.0%)	**670 (80.8%)**	28 (3.4%)	7 (0.8%)
Poultry meat	94 (11.3%)	**379 (45.7%)**	**346 (41.7%)**	10 (1.2%)
Rabbit meat	**709 (85.5%)**	112 (13.5%)	6 (0.7%)	2 (0.2%)
Pork meat	151 (18.2%)	**615 (74.2%)**	53 (6.4%)	10 (1.2%)
Eggs	60 (7.2%)	**674 (81.3%)**	82 (9.9%)	13 (1.6%)
Milk	**257 (31.0%)**	113 (13.6%)	58 (7.0%)	**401 (48.4%)**

In bold are the categories expressed most frequently for each product (Chi-Square and Goodness-of-Fit tests).

**Table 2:** Influence of demographic factors and eating habits of the participants on PC emotion (marginal means ± standard error estimated based on multivariable models including species as covariate).

		Farming System			
		Intensive		Extensive	
		Mean ± SE	*p* Value	Mean ± SE	*p* Value
Gender	Female	−0.96 ^a^ ± 0.10	**0.045**	0.88 ^a^ ± 0.05	**0.001**
	Male	−0.86 ^b^ ± 0.10		0.96 ^b^ ± 0.04	
Age	<25 years	−0.86 ± 0.12	0.135	0.93 ± 0.06	0.590
	25–40 ys	−0.84 ± 0.11		0.89 ± 0.05	
	40–55 ys	−1.06 ± 0.12		0.93 ± 0.06	
	>55 ys	−0.87 ± 0.15		0.93 ± 0.07	
Study	Primary	−0.88 ± 0.13	0.087	0.95 ^ab^ ± 0.06	**<0.001**
	Middle	−1.06 ± 0.12		0.83 ^a^ ± 0.06	
	High school	−0.82 ± 0.10		0.85 ^a^ ± 0.05	
	University degree	−0.90 ± 0.10		0.94 ^ab^ ± 0.05	
	Postgraduate	−0.88 ± 0.15		1.05 ^b^ ± 0.07	
Residence *	Village	−0.99 ^a^ ± 0.10	**0.043**	0.97 ^a^ ± 0.05	**0.003**
	Small town	−0.94 ^ab^ ± 0.09		0.94 ^a^ ± 0.04	
	City	−0.80 ^b^ ± 0.11		0.85 ^b^ ± 0.05	
Job	Student	−0.84 ± 0.12	0.054	0.95 ± 0.05	0.873
	Employee	−1.00 ± 0.10		0.92 ± 0.05	
	Artisan	−0.97 ± 0.15		0.88 ± 0.07	
	Work with animals	−0.93 ± 0.16		0.97 ± 0.08	
	Homemaker	−0.41 ± 0.01		0.96 ± 0.07	
	Freelancers/entrepreneurs	−1.11 ± 0.16		0.93 ± 0.08	
	Teachers/professors	−0.58 ± 0.21		0.81 ± 0.10	
	Unemployed	−0.63 ± 0.17		0.90 ± 0.08	
	Retired	−1.24 ± 0.17		0.94 ± 0.08	
	Other	−0.86 ± 0.28		0.93 ± 0.13	
Family structure (num. of members)	1	−1.17 ^a^ ± 0.14	**<0.001**	0.92 ± 0.06	0.970
	2	−0.71 ^b^ ± 0.12		0.93 ± 0.06	
	>2	−0.84 ^b^ ± 0.10		0.92 ± 0.05	
Animal at home	Yes	−1.30 ^a^ ± 0.17	**<0.001**	0.97 ± 0.08	0.167
	No	−0.51 ^b^ ± 0.07		0.87 ± 0.03	
Animals in the past	Never owned	−0.87 ± 0.10	0.201	0.83 ^a^ ± 0.05	**<0.001**
	Owned in the past	−0.95 ± 0.09		1.01 ^b^ ± 0.04	
Vegetarian/vegan	Yes	−0.92 ^a^ ± 0.03	**<0.001**	0.66 ^a^ ± 0.02	**<0.001**
	No	−0.80 ^b^ ± 0.01		0.94 ^b^ ± 0.01	

* Residence classification: Village (up to 5000 inhabitants); Small town (5000–30,000 inhabitants); City (over 30,000 inhabitants). Values of each parameter in the same column not sharing the same superscript (a, b) are significantly different at *p* < 0.05 (Sidak correction). Significant results (*p* < 0.05) are in bold. SE = standard error.

## Data Availability

Data are available upon request from the corresponding author.

## Supplementary Materials

The following supporting information can be downloaded at: https://www.mdpi.com/article/10.3390/vetsci10110652/s1, Figure S1: “Human Emotions and Animal Husbandry” Questionnaire; Table S1: Participants’ demographic data and eating habits; Figure S2: PC Emotion scores (means ± 95% CI) in intensive and extensive farms. PC Emotion represents a sadness-joy axis where positive scores indicate joy and hope while negative scores indicate anger, sadness, remorse/guilt, resignation, contempt/disgust, and shame/disappointment; Figure S3: Association between the perception of animal welfare conditions and the score of PC Emotion in each picture according to the farming system.
