# Supplementary material for: The Link between the Perception of Animal Welfare and the Emotional Response to Pictures of Farm Animals Kept in Intensive and Extensive Husbandry Systems: An Italian Survey
Source: Vet Sci. 2023 Nov 13;10(11):652. doi: 10.3390/vetsci10110652 (PMC10675316; doi:10.3390/vetsci10110652)

Figure S1. "Human Emotions and Animal Farming" Questionnaire.

Gender *

- ○ Male
- ○ Female

Age *

- ○ <25
- ○ 25-40
- ○ 40-55
- ○ >55

Education *

- ○ University
- ○ Middle school
- ○ High school
- ○ Elementary school
- ○ Post-graduate

Living area *

- ○ City (above 30.000 citizens)
- ○ Town (5000-30.000 citizens)
- ○ Village (up to 5000 citizens)

Profession *

- ○ Student
- ○ Employee
- ○ Workman
- ○ Working with animals (veterinarian, breeder, etc.)
- ○ Other: ______

Household members * (including yourself)

- 1
- 2
- 3
- >3

Do you have animals in your house? *

- Yes
- No

If not, please specify:

- Never had
- In the past, but not currently

For each of the following pictures:

Please, mark the intensity of each of the following emotions felt while watching at this picture, by using the corresponding scoring scale from 0 to 5, where 0=emotion not felt; 5= emotion felt at maximum intensity. *

	0	1	2	3	4	5
Anger	○	○	○	○	○	○
Joy	○	○	○	○	○	○
Sadness	○	○	○	○	○	○
Surprise	○	○	○	○	○	○
Shame / Disappointment	○	○	○	○	○	○
Resignation	○	○	○	○	○	○
Hope	○	○	○	○	○	○
Nostalgia	○	○	○	○	○	○
Remorse / Guilt	○	○	○	○	○	○
Contempt / Disgust	○	○	○	○	○	○

In your opinion, what is the level of animal welfare? *

- Very poor
- Poor
- Good
- Excellent
- Don't know

Photo 1

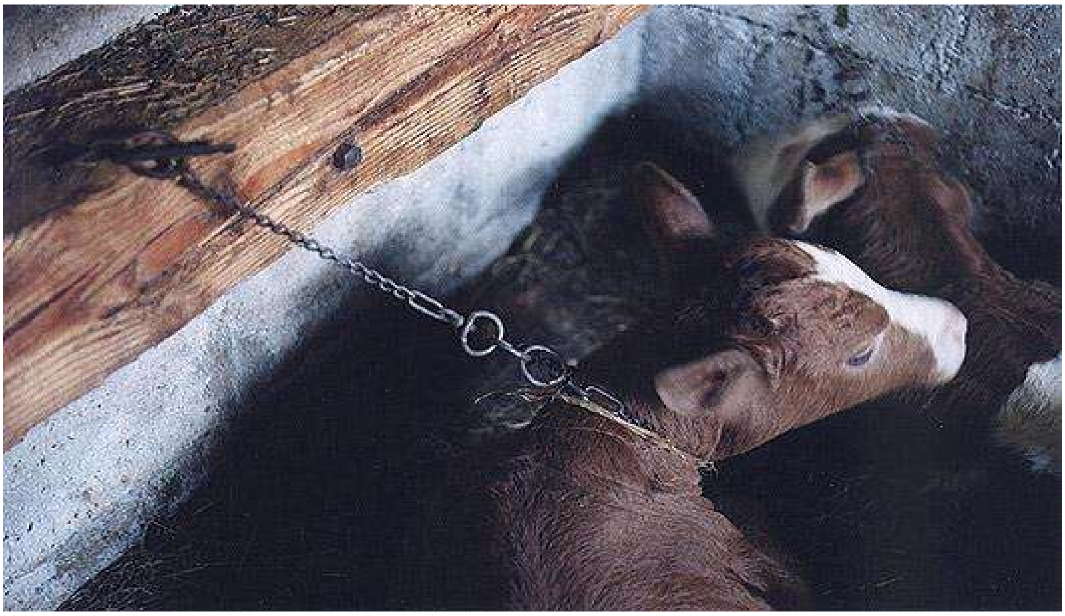

Photo 2

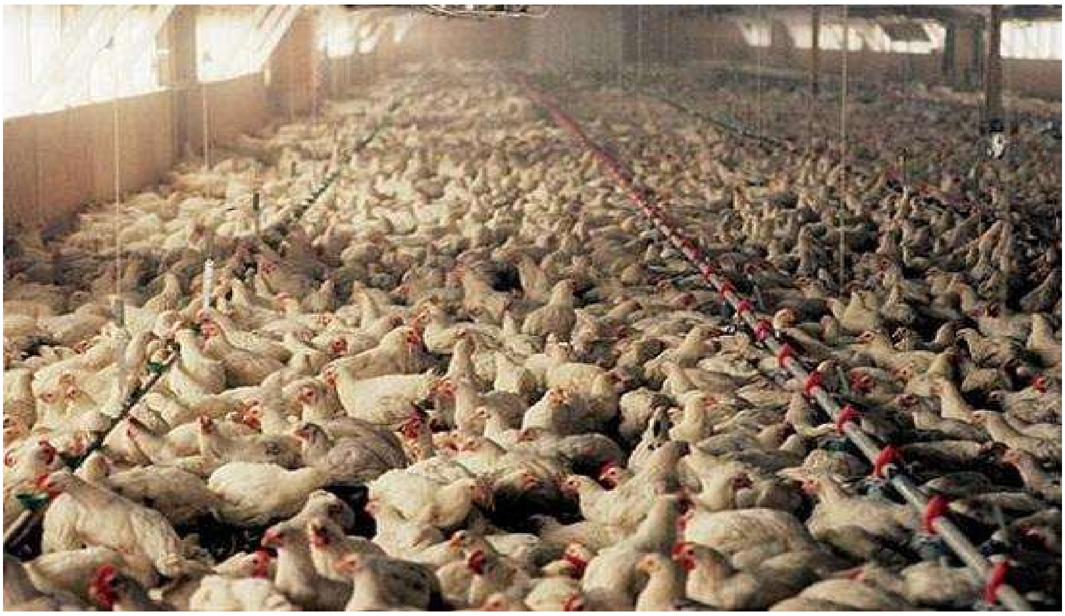

Photo 3

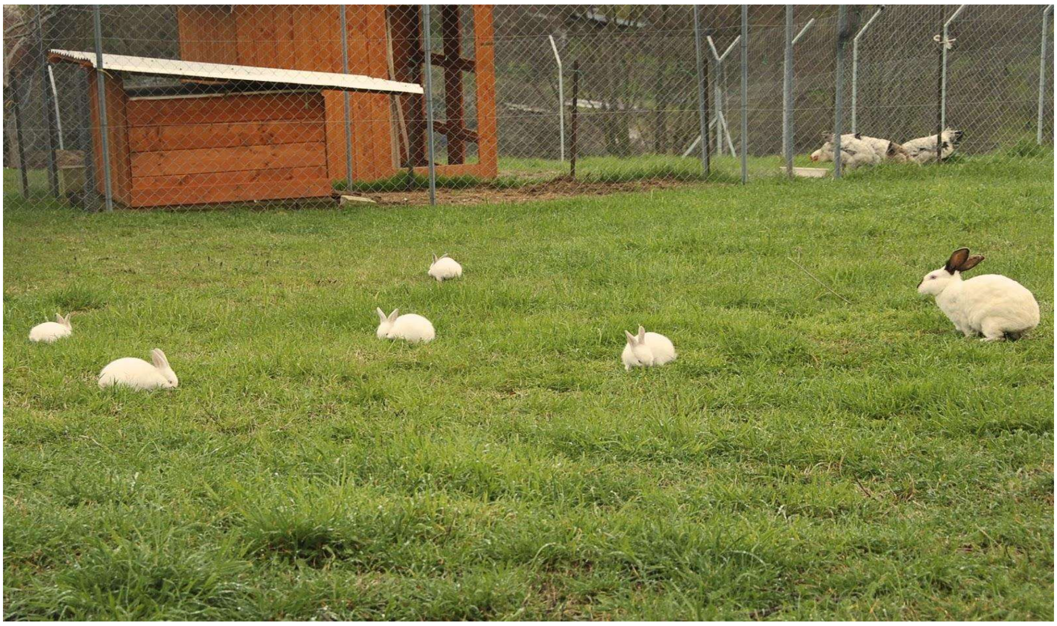

Photo 4

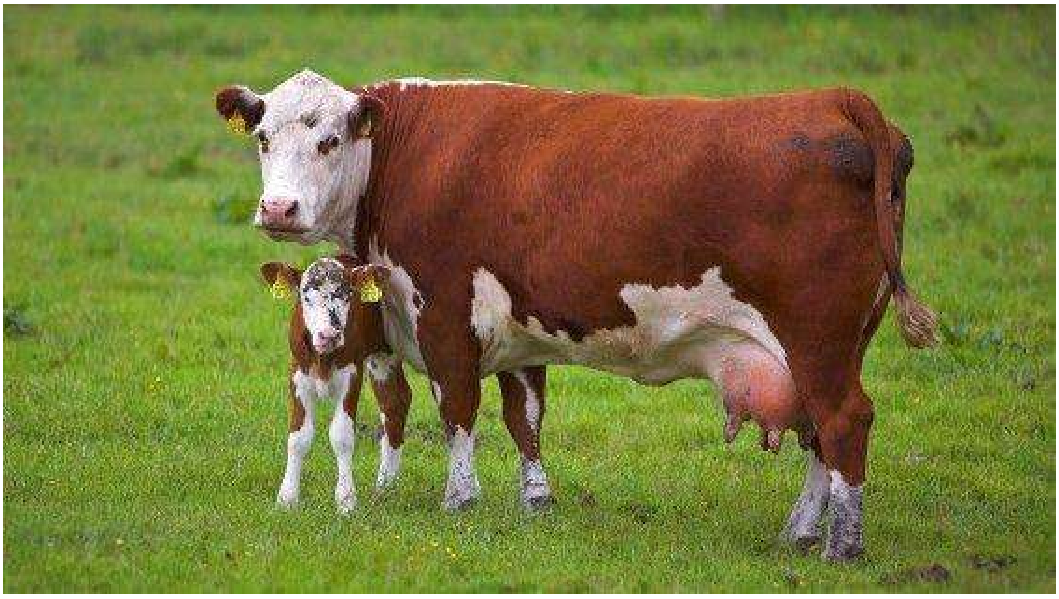

Photo 5

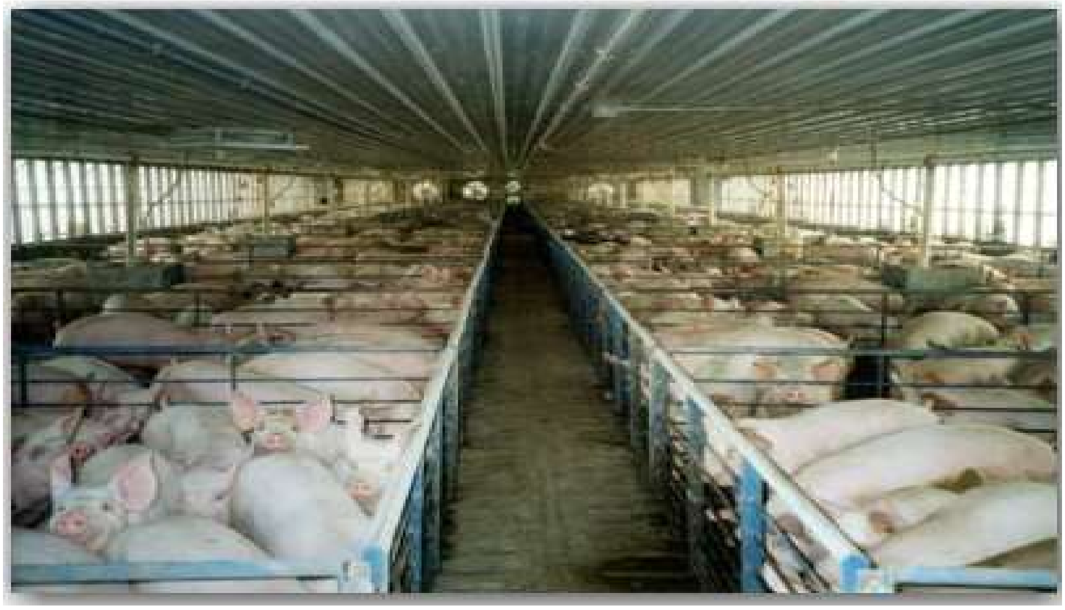

Photo 6

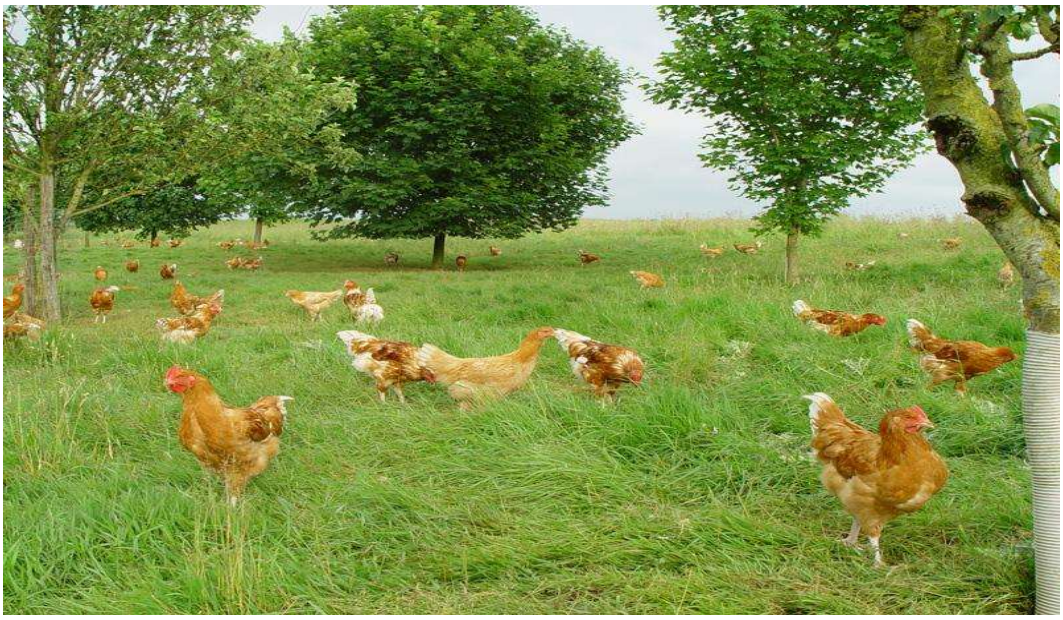

Photo 7

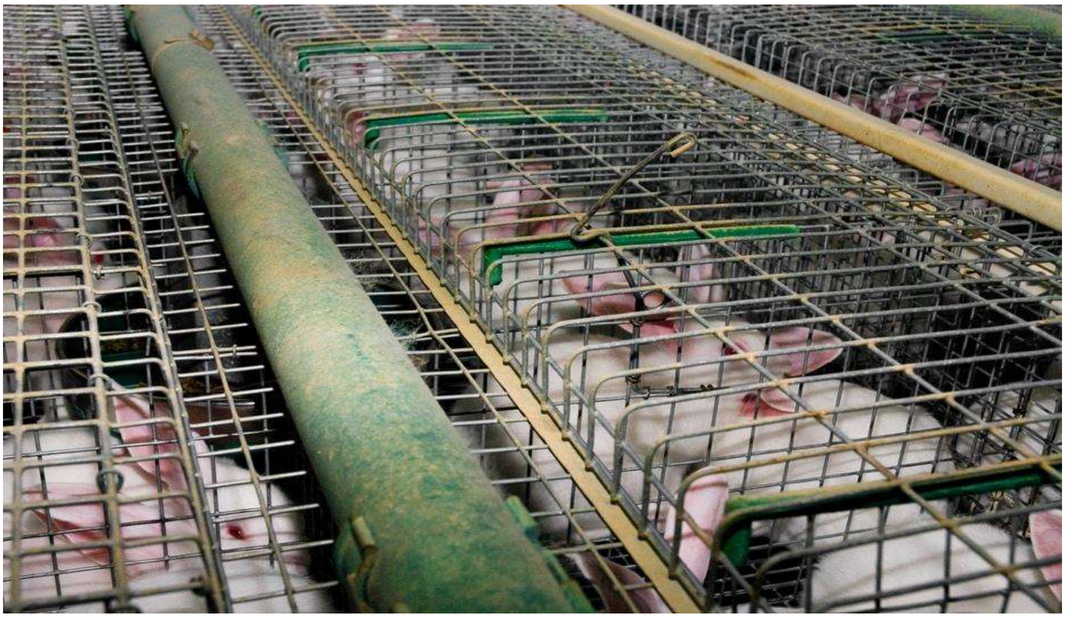

Photo 8

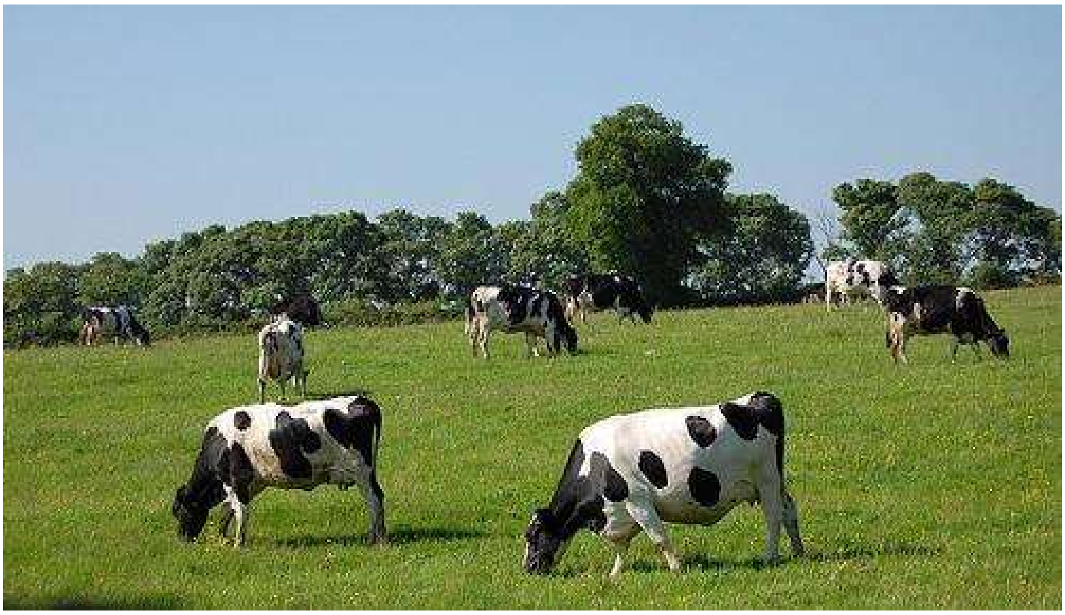

Photo 9

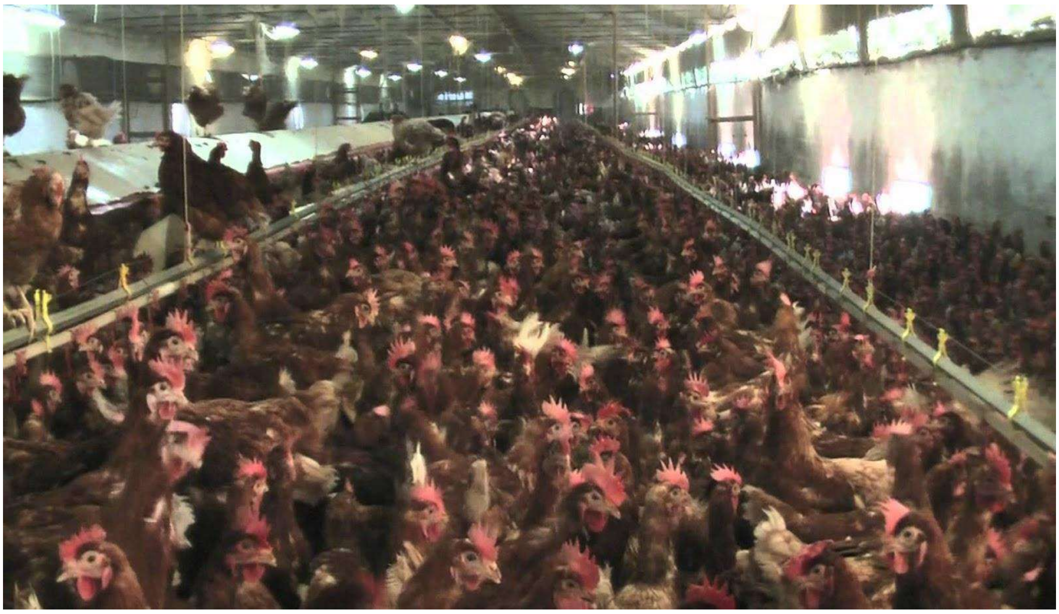

Photo 10

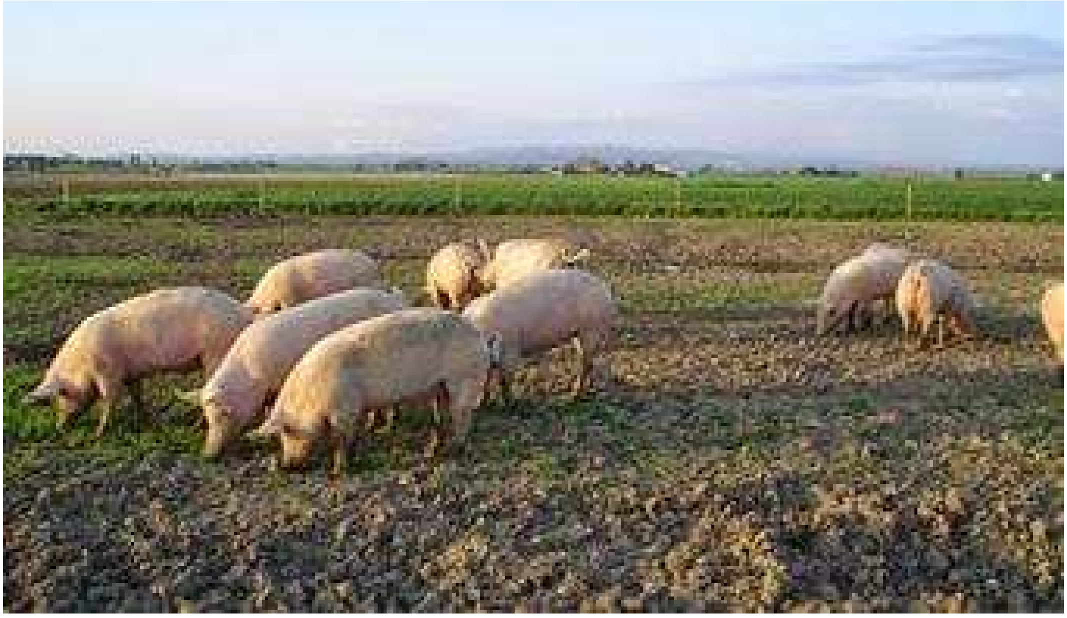

Photo 11

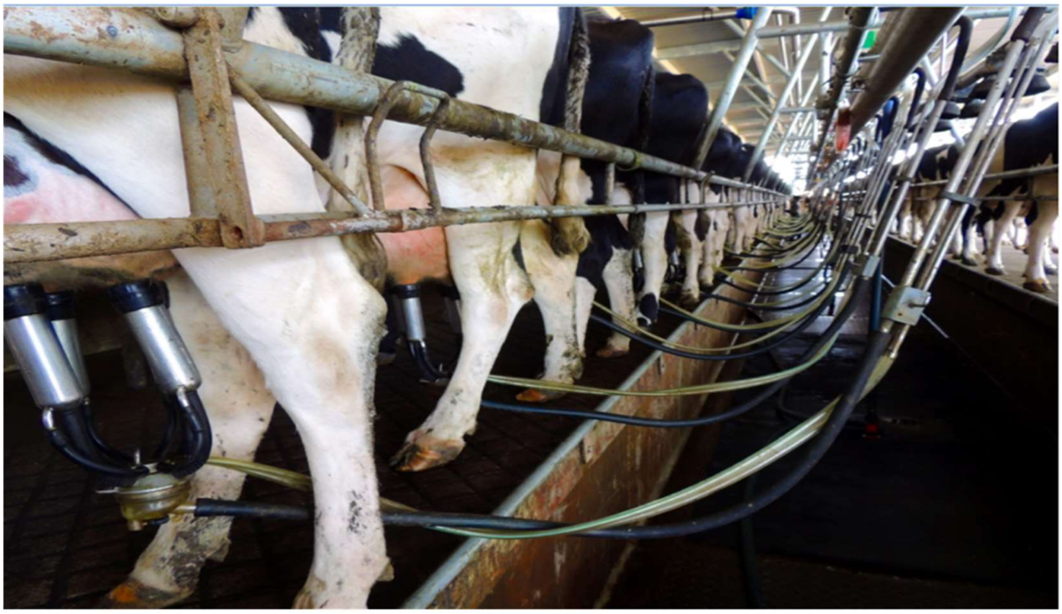

When you purchase animal products, how important is to you that the animals have been bred with good welfare standards? *

- Not important at all
- Of little importance
- Moderately important
- Important
- Very important
- Other:

How many times a week do you eat the following animal products? *

	Never	Up to 2 times a week	Up to 4 times a week	Everyday
Beef				
Chicken meat				
Rabbit meat				
Pork meat				
Eggs				
Milk				

Are you vegetarian or vegan? *

- Yes
- No

If you are, please indicate the reason beyond your choice:

- Ethical reasons
- I do not like meat
- Health reasons
- Other:

Thank you so much for your precious contribution!

Table S1. Participants demographic data and eating habits.

		Number	Percentage of respondents	P value [#]
**Gender**	**Female**	479	57.4%	<0.001
	**Male**	356	42.6%	
**Age**	**<25 years**	240	28.7%	0.007
	**25-40 years**	216	25.9%	
	**40-55 years**	170	20.4%	
	**>55 years**	209	25.0%	
**Study**	**Primary**	145	17.4%	<0.001
	**Middle**	103	12.3%	
	**High school**	360	43.1%	
	**University degree**	149	17.8%	
	**Postgraduate**	78	9.3%	
**Residence**	**Village (up to 5000 inhabitants)**	378	45.3%	<0.001
	**Small town (5000-30000 inhabitants)**	317	38.0%	
	**City (over 30000 inhabitants)**	140	16.8%	
**Job**	**Student**	237	39.4%	<0.001
	**Employe**	165	27.4%	
	**Artisan**	59	9.8%	
	**Worked somehow with animals**	57	9.5%	
	**Housewives**	5	0.8%	
	**Freelancers/entrepreneurs**	21	3.5%	
	**Teachers/professors**	13	2.2%	
	**Unemployed**	13	2.2%	
	**Retired**	28	4.7%	
	**Other**	4	0.7%	
**Family structure**	**1 member**	146	17.5%	<0.001
	**2 members**	205	24.6%	
	**>2 members**	484	58.0%	
**Animal at home**	**Yes**	639	76.5%	<0.001
	**No**	196	23.5%	
**Animals in the past ***	**Never owned**	24	19.2%	<0.001
	**Owned in the past**	101	80.8%	
**Vegetarian/vegan**	**Yes**	766	91.7%	<0.001
	**No**	69	8.3%	

[#] estimated by Chi-Square Goodness-of Fit tests. * Only for respondents who said they did not own animals at home

Figure S2. *PC Emotion* scores (means ± 95% CI) in intensive and extensive farms. *PC Emotion* represents a sadness-joy axis where positive scores indicate joy and hope while negative scores indicate anger, sadness, remorse/guilt, resignation, contempt/disgust and shame/disappointment.

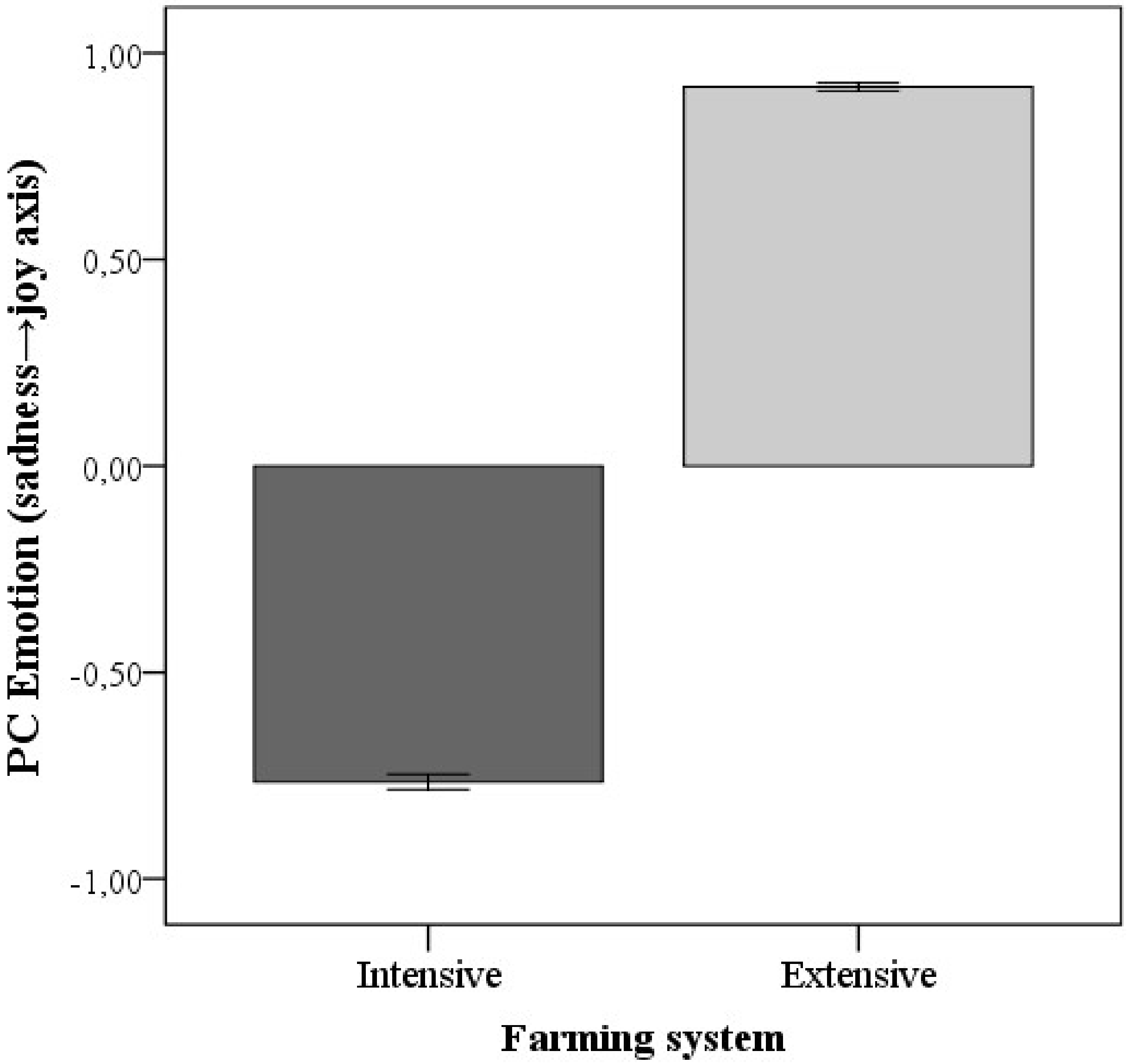

Figure S3. Association between perception of animal welfare conditions and score of *PC Emotion* in each picture according to the farming system.

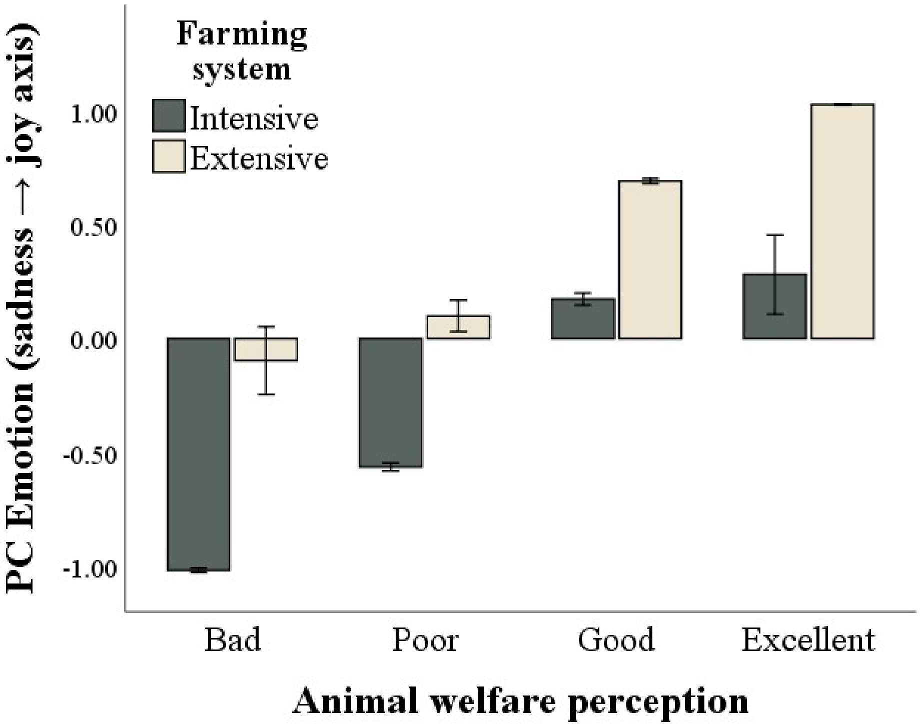

Supplement: Supplementary file 1 [file vetsci-10-00652-s001.zip › vetsci-2652895-supplementary.pdf]
